# Supplementary material for: Electrical and thermal conductivity of Earth’s iron-enriched basal magma ocean
Source: Proc Natl Acad Sci U S A. 2025 Oct 10;122(42):e2509771122. doi: 10.1073/pnas.2509771122 (PMC12557499; doi:10.1073/pnas.2509771122)
Supplement: Supplementary file 1 — Appendix 01 (PDF) [file pnas.2509771122.sapp.pdf]

Supporting Information for

Electrical and Thermal Conductivity of Earth's Iron-enriched Basal Magma Ocean

Francis Dragulet, Lars Stixrude

Corresponding Author: Francis Dragulet
E-mail: francisdragulet@g.ucla.edu

This PDF file includes:

Figs. S1 to S9
Tables S1 to S3
SI References

Table S1. Composition of simulated system

Element	Number of atoms (X_{Fe}= 0.117 / 0.5 / 1)	Oxides wt. % (X_{Fe}= 0.117 / 0.5 / 1)	Pyrolite wt. % (1)
Mg	30 / 17 / 0	38.37 / 19.24 / 0	37.80
Fe	4 / 17 / 34	9.12 / 34.29 / 59.61	8.05
Si	24	45.72 / 40.46 / 35.16	45.00
Ca	2	3.56 / 3.15 / 2.74	3.55
Al	2	3.23 / 2.86 / 2.49	4.45
O	87		

Table S2. Conductivity Fit Parameters

X_{Fe}	σ_s (S/m)	ΔE_s (kJ/mol)	ΔV_s (cm^3/mol)	σ_k (S/m)	ΔE_k (kJ/mol)	ΔV_k (cm^3/mol)
0.12	715436.899	145.342	0.263	1038523.985	117.293	0.310
0.5	515811.595	111.415	0.020	753264.805	91.485	0.089
1	310290.887	67.819	-0.122	560784.939	67.154	-0.047

Table S3. Parameters of thermal evolution model

Parameter	Symbol	Value
Mantle thermal conductivity	k_{m}	8 W/m/K
Thermal boundary layer thickness	δ	95 km
Mantle temperature	T_{m}	2730 K
Basal magma ocean specific heat	c_{m}	1000 J/kg/K
Core specific heat	c_{c}	860 J/kg/K
Core mass	M_{c}	2×10^{24} kg
Entropy of melting	ΔS	580 J/kg/K
Melting temperature at $X_{\mathrm{Fe}} = 0$	T_{A}	5650 K
Melting temperature at $X_{\mathrm{Fe}} = 1$	T_{B}	4120 K
Fe-Mg distribution coefficient	K_{D}	0.5
Core radius	b	3480 km
Rotation rate	Ω	24 hours^{-1}
Basal magma ocean adiabatic temperature gradient	∇T_{ad}	0.6 K/km
Basal magma ocean initial thickness	a_0	400 km
Basal magma ocean initial Fe-Mg fraction	$X_{\mathrm{liq},0}$	0.65

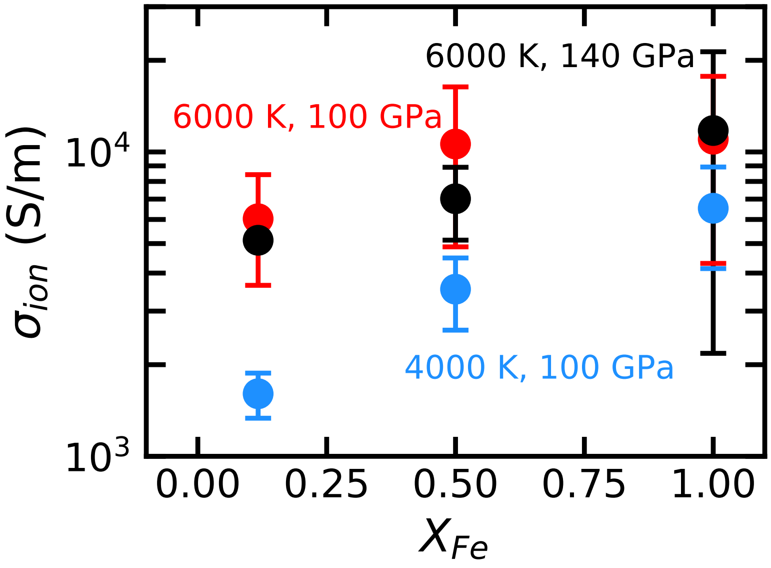

Fig. S1. Ionic conductivity σ_{ion} of silicate liquid versus iron fraction X_{Fe} = Fe/(Fe+Mg). Colors indicate different temperature and pressure conditions representative of Earth's basal magma ocean. We show results from low-spin simulations, which do not differ significantly from high-spin results.

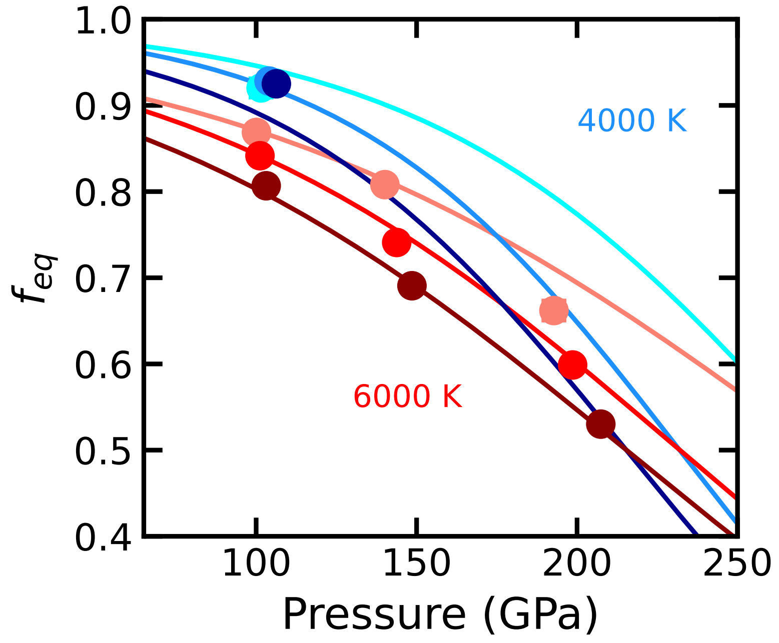

Fig. S2. Equilibrium high-spin fraction f_{eq} versus pressure, at 4000 K (blue) and 6000 K (red). Symbol darkness represents the iron fraction X_{Fe} = Fe/(Fe+Mg): light blue/red for X_{Fe} = 0.12, medium blue/red X_{Fe} = 0.5, and darker blue/red for X_{Fe} = 1. Lines are fits of isochemical data to: $f_{eq} = \left[1 + \exp\left(-\frac{E^* + PV^*}{RT}\right)\right]^{-1}$.

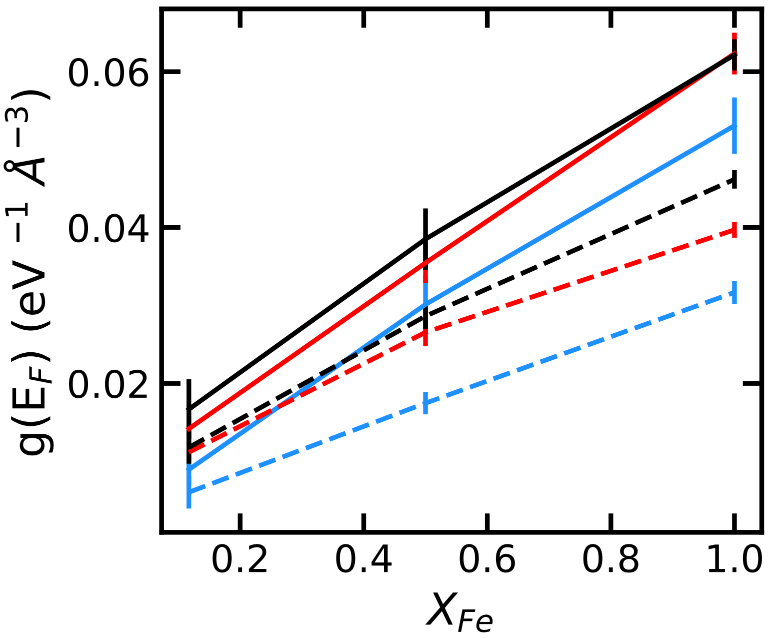

Fig. S3. Density of states at the Fermi energy, $g(E_F)$ versus iron fraction X_{Fe}. Solid lines indicate low-spin results while dashed lines represent high-spin results. Colors represent pressure and temperature conditions (blue: 4000 K and ~ 100 GPa, red: 6000 K and ~ 100 GPa, black: 6000 K and ~ 200 GPa.

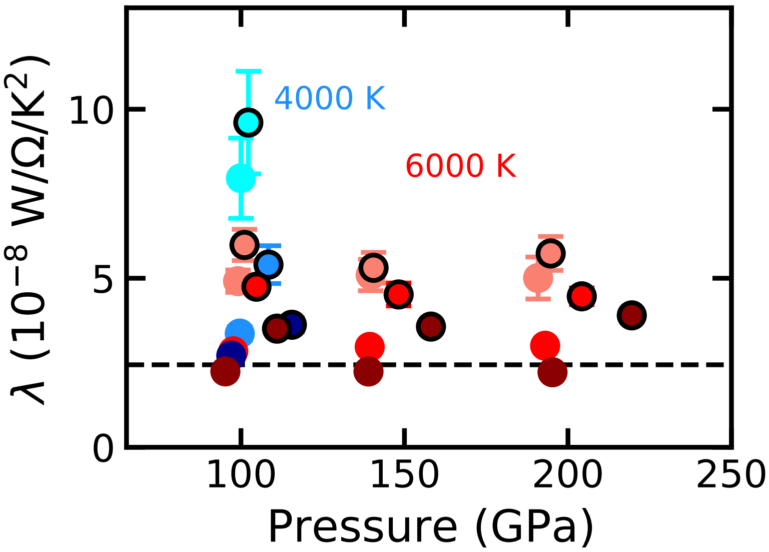

Fig. S4. Lorenz number calculated as $\lambda = k_{\text{el}}/(\sigma_{\text{el}} T)$, as a function of pressure, at 4000 K (blue) and 6000 K (red). Symbol darkness represents the iron fraction X_{Fe}: light blue/red for X_{Fe} = 0.12, medium blue/red for X_{Fe} = 0.5, and dark blue/red for X_{Fe} = 1. Symbols outlined in black denote high-spin results, while symbols without an outline represent low-spin results. The black dashed line represents the theoretical Lorenz number ($\lambda = 2.44 \times 10^{-8}$ W/Ω/K^2) typically used in applications of the Wiedemann-Franz law, determined by the free electron Sommerfeld model.

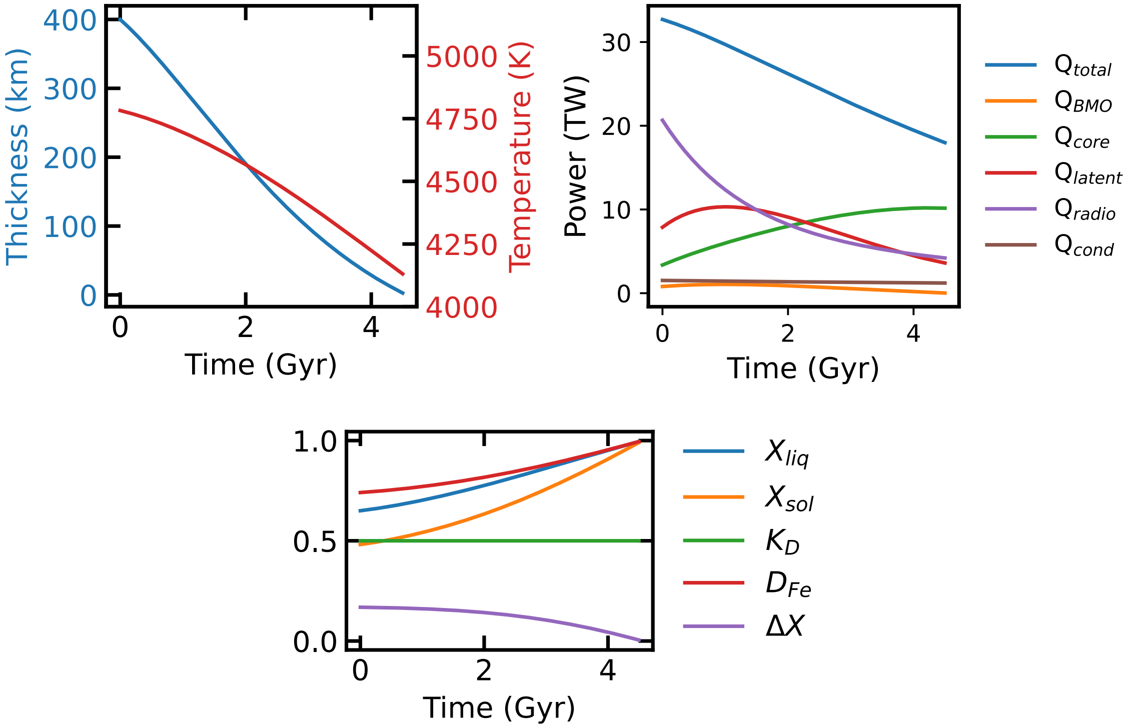

Fig. S5. Thermal evolution of the basal magma ocean. **Top Left:** thickness of the basal magma ocean (blue) and liquidus temperature (red). **Top Right:** Heat flow over time, including total outward heat flux (blue), secular cooling of the core (green) and basal magma ocean (orange), radiogenic heat (purple), latent heat of freezing (red), and conductive heat flux (brown). **Bottom:** time evolution of iron fraction in the liquid (X_{liq}) and solid (X_{sol}), Fe-Mg distribution coefficient (K_{D}), iron partition coefficient (D_{Fe}), and difference in iron fractions between solid and liquid (ΔX).

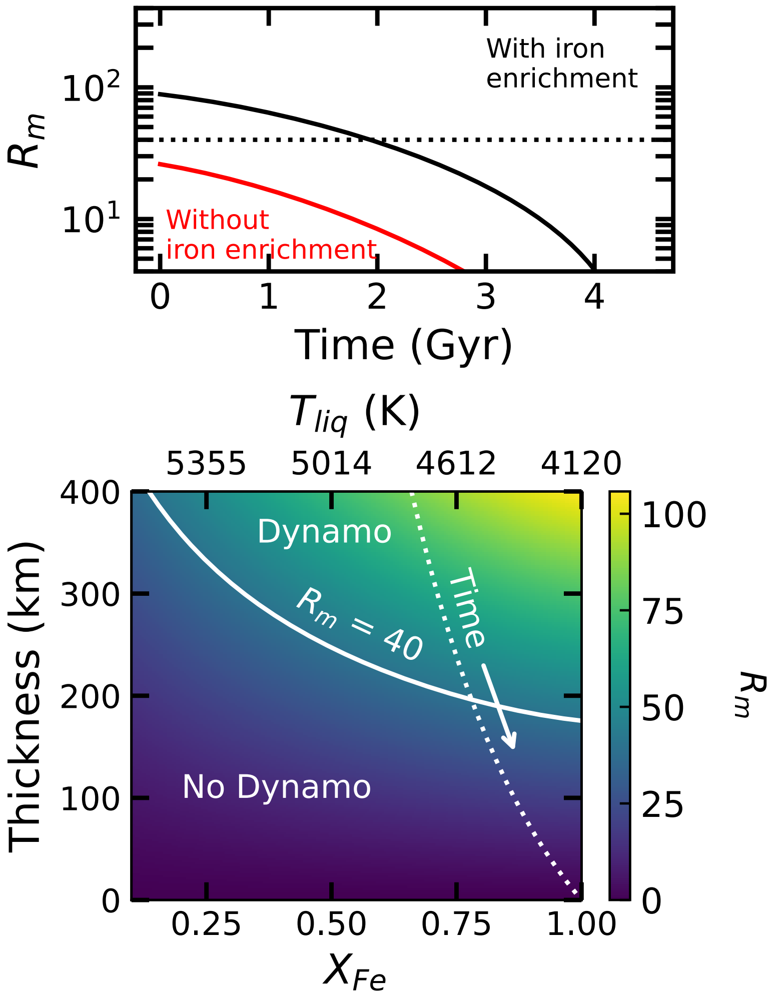

Fig. S6. Same as figure 4 in the main text, but now with Coriolis-inertial-Archimedean balance scaling for the flow velocity (equation 16).

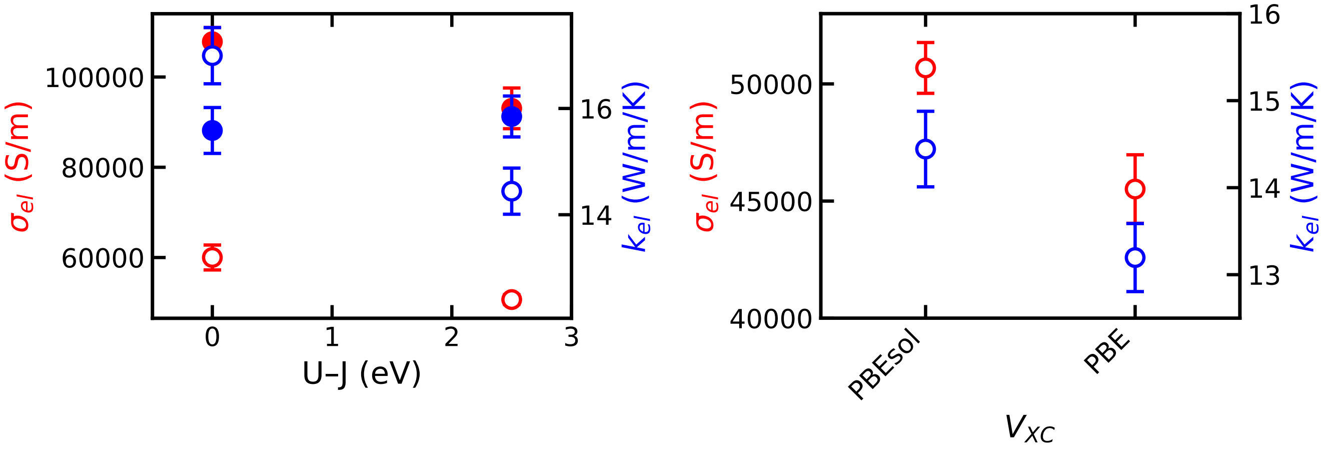

Fig. S7. Effect of U-J value (left) and exchange correlation potential V_{xc} (right) on electrical conductivity σ_{el} (red) and electronic thermal conductivity k_{el} (blue) at 6000 K, 100 GPa and $X_{\mathrm{Fe}} = 0.5$. Open circles are simulations with high-spin iron, while closed circles are with low-spin iron.

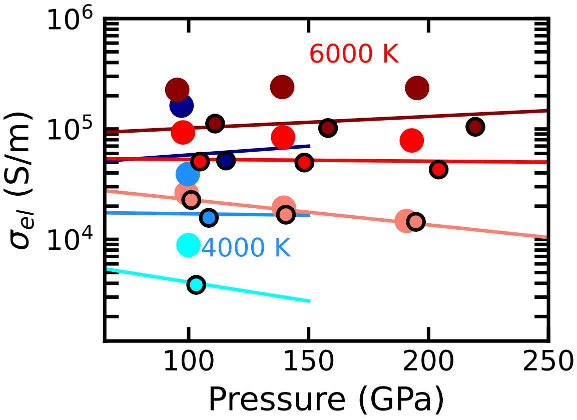

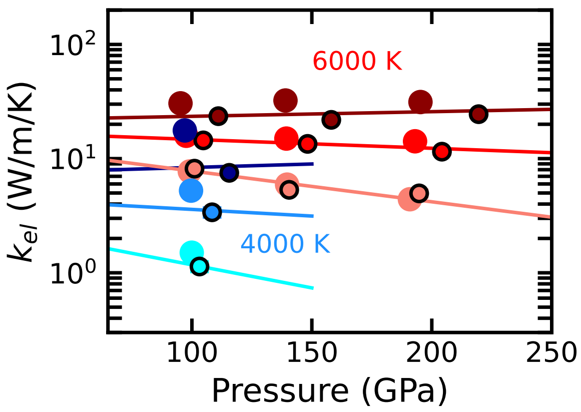

Fig. S8. Electronic contribution to electrical conductivity, σ_{el} (**left**), and thermal conductivity, k_{el} (**right**), versus pressure at 4000 K (blue) and 6000 K (red). Symbol darkness represents the iron fraction X_{Fe}: light blue/red for X_{Fe} = 0.12, medium blue/red for X_{Fe} = 0.5, and dark blue/red for X_{Fe} = 1. Larger symbols denote low-spin results, while smaller symbols outlined in black denote high-spin results. Lines are fits of data at constant X_{Fe} and spin state to equations 8 and 9 in the main text.

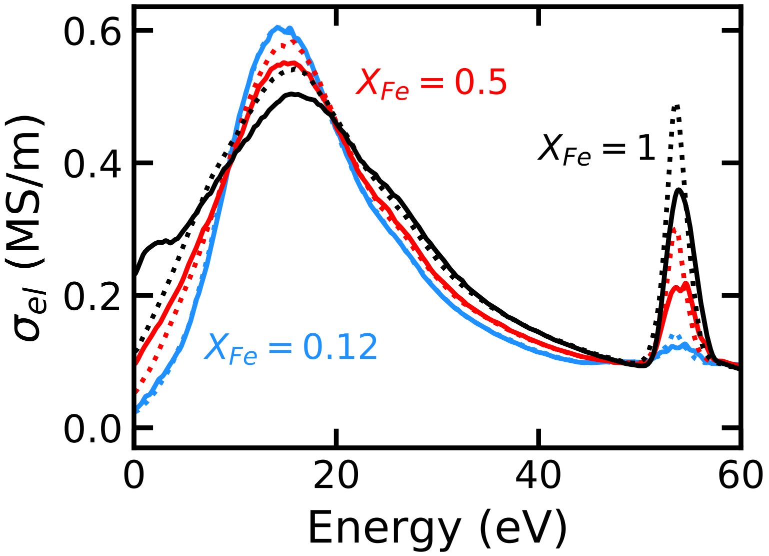

Fig. S9. Electronic electrical conductivity as a function of energy at 6000 K and 100 $\pm$ 10 GPa for various iron fractions (colors), and spin states (solid: low-spin, dotted: high-spin).

References

1. WF McDonough, SS Sun, The composition of the Earth. *Chem. geology* **120**, 223–253 (1995).
